# Transfusional Malaria and Associated Factors at the National Blood Transfusion Center of Niamey-Niger

**DOI:** 10.1155/2019/7290852

**Published:** 2019-04-01

**Authors:** Aminata Iro, Moustapha Mahamane Lamine, Ramatoulaye Hamidou Lazoumar, Ibrahim Alkassoum, Daou Maman, Harouna Amadou Mahaman Laouali, Mahamadou Doutchi, Seydou Maiguizo, Ibrahim Maman Laminou

**Affiliations:** ^1^Faculté de Science de la Santé de l'Université de Niamey, Niger; ^2^Université Check Anta Diop de Dakar, Senegal; ^3^Centre de Recherche Médicale et Sanitaire, Niger; ^4^Hôpital National de Niamey, Niger; ^5^Université de Maradi, Niger; ^6^Université de Zinder, Niger; ^7^Centre National de Transfusion Sanguine de Niamey, Niger

## Abstract

**Summary:**

* Problem*. Transfusional malaria is an accidental transmission of* Plasmodium* via a blood transfusion. Its magnitude is underestimated and very little data on the assessment of this risk are available in Niger.

**Objective:**

This study aimed to determine the prevalence of plasmodial infection of blood bags at the National Blood Transfusion Center of Niamey (NBTC).

**Methodology:**

A cross-sectional study to diagnose* Plasmodium* infection by microscopy and Rapid Diagnostic Test (RDT) was carried out during the rainy season (September to November 2015). Blood grouping was performed by the BETH-VINCENT technique.

**Results:**

One thousand three hundred and fifty-seven (1357) blood bags were collected. One hundred and fifty-seven (11.6%) of the donors were infected with* Plasmodium* by microscopy and 2.4% (9/369) by rapid diagnostic test. All infections were with* P. falciparum* (100%). The mean parasite density was 197 parasites/*μ*L (SD=281; [80: 2000]). There were no significant differences in infection prevalence between the ABO blood groups (*p* = 0.3) or the rhesus positivity (*p=08*). There is also no significant difference in temporal (*p* = 0.1) and spatial (*p* = 0.6) distribution.

**Conclusion:**

The transmission of transfusional malaria during the rainy season is a fact in Niger. Such risks were independent of the ABO blood type and positivity for the rhesus antigen. Pretransfusion diagnosis or posttransfusion therapy should be instituted to prevent it.

## 1. Introduction

Vector transmission of malaria is the most common mode of transmission. However, malaria is also rarely transmitted vertically from mother to child during pregnancy and accidentally by blood transfusion. The transfusion of blood that began in 1910 [1] has now become a very common act. In Niger, 98000 blood bags were collected at the Niamey National Blood Transfusion Center in 2016 [2]. This blood is mainly intended for maternity and pediatric and surgical services, as well as for National Center for Sickle Cell Diseases and medical emergencies, where the demand of blood is relatively high. The transfusional transmission of malaria exists and should be described in malaria-endemic countries, particularly in high-transmission seasons.

In Niger, few data exist on the risk of transfusional transmission of malaria. Transfusional malaria is particularly severe in pregnant women, children under five, naïve travellers, and immune-compromised patients. It is even often lethal [3]. As recommended by the World Health Organization (WHO), infectious diseases such as HIV, hepatitis B, hepatitis C, and syphilis are screened at the National Blood Transfusion Center [4, 5]. On the other hand, the screening of donors is not systematically done for parasitic infections such as malaria, trypanosomiasis, toxoplasmosis, or babesiosis. In sub-Saharan Africa, 40% of donated bloods are often not screened for transfusion transmissible infections such as malaria [6]. The prevalence of* Plasmodium* contaminated blood varies from 6.5% to 74.1% depending on the area [7]. Indeed, the asymptomatic carriage of* Plasmodium* in blood donors varies according to the epidemiological context of malaria and especially the intensity of transmission of the area. This prevalence is 33.5% in Benin [8], 29.5% in Guinea [7], 28.3% in the DRC Gongo [6], 23% in Nigeria [7], 4, 1% in Ethiopia [9], 3% in Ghana [10], 1.3% in Uganda [11], 1.4% in Bamako in Mali [12], and none in Egypt [13].

It is in this context that we conducted this study to evaluate the prevalence of plasmodial infection of donated blood at the National Blood Transfusion Center of Niamey in Niger.

## 2. Methodology

### 2.1. Study Design

A cross-sectional study was set up during the raining season, from September to November 2015, to determine the prevalence of plasmodial infection of donors at Niamey National Blood Transfusion Center, which is the National Reference Center (CNR) for the production of blood components.

### 2.2. Sampling and Population

The study population consisted of volunteer blood donors. Donors were selected according to the criteria of medical selection. The inclusion of criteria was (1) absence of fever, (2) absence of clinical signs of disease, (3) having a weight more than 50 kg, (4) age having to be between 17 and 65 years, and (5) the date of last donation having to be 4 months for women and 3 months for men. The criteria of noninclusion are (1) having an age under 17 years, (2) having a fever, (3) having a clear sign of illness (HIV, sickle cell), (4) being in a state of pregnancy, (5) having menstrual cycles, and (6) refusing to give consent to study. The sample size of 184 donors was estimated by using an estimated prevalence of 20% of infection, 95% confidence interval, and an accuracy of 5%.

### 2.3. Collection of Samples

The collection was done by questionnaire to collect all the useful information (identification number, age, sex, origin, blood group, rhesus etc…). The study samples were collected from volunteer blood donors aged 18-65 years. The blood samples were collected in 5 ml tubes containing EDTA (Ethylene Diamine Tetra Acetic). Thick and thin blood smears were prepared for microscopy diagnosis of malaria that followed a rapid diagnostic test.

### 2.4. Laboratory

#### 2.4.1. Rapid Diagnostic Test of Malaria

On each blood bag of donors was carried out a rapid diagnostic test of malaria. The rapid diagnostic test used is SD Bioline®. The test detects specific malarial antigens: HRP II.

#### 2.4.2. Confection of Thick and Thin Smear

The biological confirmation of plasmodial infection was by thick and thin smears microscopy, which is the gold standard for malaria diagnosis. The thick blood smears were made from 10 *μ*l of venous blood. With the corner of another blade, the drop of blood is spread slightly in circular motions until the bloodstain is evenly thickened. This mechanical defibrination technique takes about 15 seconds allowing spreading of the blood in a circular surface. The slide was dried for about two hours at laboratory temperature.

The thin smear is a thin layer of blood spread on the surface of a slide. This examination is performed to determine the plasmodial species. 5 *μ*l of venous blood collected on EDTA tube is deposited on the slide. The edge of a second blade is then placed in front of the drop of blood, tilted to 45°, and pulled forward in a fast and regular gesture towards the free end of the slide carrying the drop of blood. Thin smears are fixed with alcohol (methanol) before staining.

#### 2.4.3. Slide Staining

The slides were stained with May Grunwald Giemsa diluted at 1/10 for 20 minutes, rinsed with buffered water at pH = 7.2, and allowed to dry at room temperature.

#### 2.4.4. Microscopic Reading

Reading of the blood smears was made using a binocular 100X objective immersion microscope by experienced technicians. Parasitaemia was determined by the quantitative leukocyte method. The number of parasites (N) is counted at the same time as the leucocytes on the slide. The counting was stopped after reaching a total of 200 leucocytes for each slide. The parasitaemia (P) was then calculated using the following formula: parasitaemia = 8000XN / 200, where N is the number of parasites counted on 200 leucocytes under the microscope and 8000 is the average number of leucocytes per *μ*l of blood in adults. Slides were considered negative if no parasites were detected following a full scan of the smear.

### 2.5. Ethics

The Faculty of Health Science Committee approved this study. Patient's consent and anonymity were respected throughout the study.

### 2.6. Statistical Analysis

The data was captured and analysed using EPI INFO software version 7.0. Chi square test was used to compare prevalence.* p values* less or equal to 0,05 are considered significant.

## 3. Results

### 3.1. Characteristics of the Population

One thousand three hundred and fifty-seven (1357) donors' bloods were collected and examined for* Plasmodium* infection between September and November 2015. The average age of blood donors was 31.9 years (SD = 10.1, [24; 38]). The median is 30. The average age of the most frequent donors was 25 years. The male-female sex ratio was 3.6.

Ninety-one point thirty-five percent (N = 1226) of the blood donors were from the urban community of Niamey. The urban community of Niamey (91.4%) and Torodi (1.3%) and the department of Kollo (1%) are the largest purveyors of blood bags at CNTS Niamey. Six hundred and nine (44.9%) donors were sampled and reviewed in September against 671 (49.6%) in October and 77 (5.7%) in November 2015.

### 3.2. Prevalence of* Plasmodium falciparum* Infection

Eleven point six percent (11.6%) of blood donors were infected with* Plasmodium*. Eighty-eight point four percent (88.4%) were usable because not infected. The mean parasite density was 197 parasites/*μ*L (SD=281, [80; 2000]). Two point four percent (9/369) are infected by RDT. All infections are* Plasmodium falciparum* (100%).

### 3.3. Distribution of* Plasmodium* Infection by Blood Group and Rhesus Antigen

Table 1 shows the distribution of infections by blood group.

There is no statistically significant difference in infection by blood group (*p* = 0.3).

Seventeen point five percent (17.5%) of RH D negative donors were infected (17/97) versus 11.1% (140/1260) of RH D positive donors. However, there was no difference in the infection prevalence by rhesus antigen (*p* = 0.05).

Table 2 shows the distribution of* Plasmodium* infections by blood groups (ABO) and rhesus antigen (RH D).

There was no statistically significant difference in infection by blood group (*p* = 0.08).

### 3.4. Distribution of* Plasmodium* Infection by Donor Age and Sex

Dividing* Donors* into* Two Age Groups.* Those under the median (30 years) and those over the median resulted in 11.6% (82/157) infections in the donors under 30 and 11.5% (75/157) in the over 30-year age group. There was no statistical difference in plasmodial infection by age group.

Seventy five point fifty-two percent (117/1060) of male donors were positive for* P. falciparum* versus 25.5% (40/297) of female donors. There was no statistically significant difference in the infection by sex of donors (*p* = 0.24).

### 3.5. Distribution of Infection by Sampling Period and Patient Provenance

The temporal distribution of infection shows that there are 12.3% (75/157) of donors that are infected in September, 11.6% (78/157) in October, and 5.2% in November (4/157). There is no statistically significant difference in temporal distribution of infections (*p* = 0.1).

The spatial distribution of infections shows that 14.3% (2/12) of Kollo donors, 11.9% (146/1080) of donors from Niamey, and 5.9% of Torodi donors (1/16) carry* Plasmodium*. There is no statistically significant difference in spatial distribution of infections (*p* = 0.6).

## 4. Discussion

This cross-sectional study assesses the prevalence of* Plasmodium* infection by microscopy and RDT in blood donors at the National Blood Transfusion Center in Niamey, Niger. This study was conducted by the Parasitology Unit of the Centre de Recherche Médicale et Sanitaire (CERMES) in collaboration with the National Blood Transfusion Center of Niamey from September to November 2015.

Eleven point six percent (11.6%) of donors were infected with* Plasmodium* by microscopy and 2.4% (9/369) by RDT. A meta-analysis conducted in sub-Saharan Africa shows that the prevalence of* Plasmodium*-contaminated blood varied from 6.5% to 74.1% in different areas [7]. In fact, the asymptomatic carriage of* Plasmodium* is very common in tropical Africa and varied according to the intensity of transmission. Thus, the prevalence of parasite carriage in blood donors is 33.5% in Benin [8], 29.5% in Guinea [7], 28.3% in DRC Congo [6], 23% in Nigeria [7], 4.1% in Ethiopia [9], 3% in Ghana [10], 1.4% in Bamako in Mali [12], 1.3% in Uganda [11], and none in Egypt [13].

All infections were* Plasmodium falciparum* (100%), which is the most virulent* Plasmodium* species known till date. No infection with* Plasmodium malaria*,* Plasmodium ovale*, and* Plasmodium vivax* was found in Niger. Many studies have shown that* Plasmodium falciparum* is the most commonly transmitted species during blood transfusions [14]. In Benin,* Plasmodium falciparum* accounts for 96.6% of infections while* Plasmodium ovale* and* Plasmodium malariae* account for only 3.3% [8]. In Uganda, the plasmodial species encountered are* Plasmodium falciparum* (80%) and* Plasmodium malaria* (20%) [11]. In Ethiopia, the species found in blood donors are 52.9% for* Plasmodium vivax* and 47.1% for* Plasmodium falciparum* [9] with parasite densities between 100 and 500 P/*μ*L. Finally, the prevalence of* Plasmodium falciparum* infection is 2% in Group O donors who are the most infected in Bamako, Mali [12].

There is no difference of the infection by blood type (*p* = 0.3) and blood group (*p* = 0.05) infection, such as that described by Ibhanesebhor [15]. The ABO system is not associated with the incidence of uncomplicated malaria infection but rather with the formation of rosettes [16]. However, some studies have shown that group O subjects are more likely to be infected with* Plasmodium* [9] and that even multiple infections are more common in-group O subjects [17]. The relative proportion of group O subjects in a general population explains this. There is no risk of transfusion malaria due to sex (*p*=0.24) or age of donors. There is also no statistically significant difference in either temporal (*p*=0.1) or spatial (*p*=0.6) distribution of infections. However, a study in Mali over a 12-month period shows that the main risk factor is the transmission season during which the prevalence of infection among blood donors is highest [12].

Several measures of prevention of transfusion malaria can be envisaged according to the epidemiological situation of malaria and especially its transmission intensity.

In malaria-free zones such as France and Canada preblood donation interviews to avoid a “risky” candidate, such as returning immigrants or donors who have stayed in a malaria-endemic area from blood donation, can be put in place [3]. Screening of blood donors by antibody detection methods such as immunofluorescence (*Plasmodium* SPOT IF) and ELISA can also be used even if the presence of antibodies does not indicate a contaminating power [18, 19]. Finally, a recent study by a Cambridge research team shows that treating blood with both UV and vitamin B reduces the risk of malaria transmission during blood transfusions [20].

In endemic malaria areas such as Niger, diagnosis by rapid diagnostic test (RDT) is possible. However, RDTs have major disadvantages of being very insensitive to detect low parasite densities [4]. Also, parasite carriage is very common in sub-Saharan Africa, which may eliminate many blood donors and increase the risk of death by severe anaemia of pregnant women and children less than five years of age [21]. In sub-Saharan Africa, the majority of the population is semi-immune to malaria. The question that arises is as follows: what is the risk of transmitting some additional parasites to a semi-immune chronic carrier? Pregnant women, children under five, and immune-compromised, travellers from malaria-free countries are most at risk of contracting transfusion malaria because studies have shown that a few parasites are enough to trigger severe malaria [21]. Microscopy diagnosis allows the detection of the infection. The detection threshold is about 10 to 20 parasites / *μ*L [22]. An algorithm including TDR examination and microscopy if TDR is positive would be very useful. However, microscopy is difficult to set up systematically because it is time-consuming and highly dependent on the experience of the technician. Serology (IFI, ELISA) is almost always positive in endemic malaria because of the frequent asymptomatic carriage and will result in the exclusion of donors who are no longer infected or the risk of making unusable the few collections of donated blood samples [23]. It is more indicated in malaria free countries. Because of submicroscopic infections, molecular biology tools such as polymerise chain reaction (PCR) and Reverse Transcriptase Polymerise Chain Reaction (RT-PCR) can be used to detect* Plasmodium* [24, 25]. However, these techniques are expensive, inaccessible routinely in malaria-endemic countries, and require adequate training. In areas of stable malaria transmission, the use of long lasting insecticide treated nets (LLINs) coupled with retention of blood donors may be an alternative [6]. Transfused patients may also benefit from presumptive antimalarial treatment with artemisinin-based combined therapy [5, 8, 21]. Currently this strategy is the most used in developing countries because it has the best cost-benefit ratio. Finally, posttransfusion screening of transfused patients can be used during clinical investigations to assess the extent of transfusion-transmitted malaria [26].

The main limitation of this study is in the nonuse of more sensitive diagnostic tools to identify sub microscopic infections.

## 5. Conclusion

The risk of transfusional malaria is relatively high in Niger. The infection is not associated with blood types, groups, gender, age, geographic origin, or donor sampling period. Post transfusion treatment of patients with antimalarial and preblood donation screening may be a way to prevent transfusional malaria in Niger.

## Figures and Tables

**Table 1:** Distribution of *Plasmodial* infections by blood type.

Blood Group	Infection present	Infection absent	Total	% infected
A	38	266	304	12.5
AB	2	47	49	4.1
B	34	285	319	10.7
O	83	602	685	12.1

**Table 2:** Relationship between blood infection and blood group.

Blood group	Infection present	Infection absent	Total	% infected
A-	7	21	28	25
A+	31	245	276	11.2
B-	1	19	20	5
B+	33	266	299	11
AB-	0	6	6	0
AB+	2	41	43	4.7
0-	9	34	43	20.9
0+	74	568	642	11,5

## Data Availability

The data used to support the findings of this study are available from the corresponding author upon request.
